# From mouse to mouse‐ear cress: Nanomaterials as vehicles in plant biotechnology

**DOI:** 10.1002/EXP.20210002

**Published:** 2021-08-17

**Authors:** Xue Xia, Bingyang Shi, Lei Wang, Yang Liu, Yan Zou, Yun Zhou, Yu Chen, Meng Zheng, Yingfang Zhu, Jingjing Duan, Siyi Guo, Ho Won Jang, Yuchen Miao, Kelong Fan, Feng Bai, Wei Tao, Yong Zhao, Qingyu Yan, Gang Cheng, Huiyu Liu, Yan Jiao, Shanhu Liu, Yuanyu Huang, Daishun Ling, Wenyi Kang, Xue Xue, Daxiang Cui, Yongwei Huang, Zongqiang Cui, Xun Sun, Zhiyong Qian, Zhen Gu, Gang Han, Zhimou Yang, David Tai Leong, Aiguo Wu, Gang Liu, Xiaogang Qu, Youqing Shen, Qiangbin Wang, Gregory V. Lowry, Ertao Wang, Xing‐Jie Liang, Jorge Gardea‐Torresdey, Guoping Chen, Wolfgang J. Parak, Paul S. Weiss, Lixin Zhang, Martina M. Stenzel, Chunhai Fan, Ashley I. Bush, Gaiping Zhang, Christopher P. L. Grof, Xuelu Wang, David W. Galbraith, Ben Zhong Tang, Christina E. Offler, John W. Patrick, Chun‐Peng Song

**Affiliations:** ^1^ Henan‐Macquarie University Joint Centre for Biomedical Innovation, School of Life Sciences Henan University Kaifeng Henan China; ^2^ Henan Key Laboratory of Brain Targeted Bio‐nanomedicine, School of Life Sciences & School of Pharmacy Henan University Kaifeng Henan China; ^3^ State Key Laboratory of Crop Stress Adaptation and Improvement Henan University Kaifeng Henan China; ^4^ School of Environmental and Life Sciences, College of Engineering, Science and Environment University of Newcastle Callaghan New South Wales Australia; ^5^ Department of Biomedical Sciences, Faculty of Medicine and Health Sciences Macquarie University Sydney New South Wales Australia; ^6^ Materdicine Lab, School of Life Sciences Shanghai University Shanghai China; ^7^ School of Energy and Power Engineering Nanjing University of Science and Technology Nanjing China; ^8^ Department of Material Science and Engineering, Research Institute of Advanced Materials Seoul National University Seoul Republic of Korea; ^9^ Engineering Laboratory for Nanozyme, Institute of Biophysics Chinese Academy of Sciences Beijing China; ^10^ Key Laboratory for Special Functional Materials of Ministry of Education, National & Local Joint Engineering Research Center for High‐efficiency Display and Lighting Technology, School of Materials Science and Engineering, Collaborative Innovation Center of Nano Functional Materials and Applications Henan University Kaifeng Henan China; ^11^ Center for Nanomedicine and Department of Anesthesiology, Brigham and Women's Hospital Harvard Medical School Boston Massachusetts USA; ^12^ School of Materials Science and Engineering Nanyang Technological University Singapore Singapore; ^13^ Beijing Advanced Innovation Centre for Soft Matter Science and Engineering, State Key Laboratory of Organic‐Inorganic Composites, Bionanomaterials & Translational Engineering Laboratory, Beijing Laboratory of Biomedical Materials Beijing University of Chemical Technology Beijing China; ^14^ Centre for Materials in Energy and Catalysis (CMEC), School of Chemical Engineering and Advanced Materials The University of Adelaide Adelaide South Australia Australia; ^15^ College of Chemistry and Chemical Engineering Henan University Kaifeng Henan China; ^16^ Advanced Research Institute of Multidisciplinary Science, School of Life Science, Institute of Engineering Medicine, Key Laboratory of Molecular Medicine and Biotherapy Beijing Institute of Technology Beijing China; ^17^ Institute of Pharmaceutics, Zhejiang Province Key Laboratory of Anti‐Cancer Drug Research, Hangzhou Institute of Innovative Medicine Zhejiang University Hangzhou China; ^18^ State Key Laboratory of Medicinal Chemical Biology, College of Pharmacy Nankai University Tianjin China; ^19^ Institute of Nano Biomedicine and Engineering, Key Laboratory for Thin Film and Microfabrication Technology of the Ministry of Education, Shanghai Engineering Research Center for Intelligent Diagnosis and Treatment Instrument, Department of Instrument Science & Engineering, School of Electronic Information and Electrical Engineering Shanghai Jiao Tong University Shanghai China; ^20^ Laboratory for NanoMedical Photonics, School of Basic Medical Science Henan University Kaifeng Henan China; ^21^ State Key Laboratory of Virology, Wuhan Institute of Virology, Center for Biosafety Mega‐Science Chinese Academy of Sciences Wuhan China; ^22^ College of Materials Science and Engineering Sichuan University Chengdu China; ^23^ State Key Laboratory of Biotherapy and Cancer Center, National Clinical Research Center for Geriatrics, West China Hospital Sichuan University Chengdu China; ^24^ College of Pharmaceutical Sciences Zhejiang University Hangzhou China; ^25^ Department of Biochemistry and Molecular Pharmacology University of Massachusetts Medical School Worcester Massachusetts USA; ^26^ State Key Laboratory of Medicinal Chemical Biology, Key Laboratory of Bioactive Materials, Ministry of Education, College of Life Sciences Nankai University Tianjin China; ^27^ Department of Chemical and Biomolecular Engineering National University of Singapore Singapore Singapore; ^28^ Cixi Institute of Biomedical Engineering, International Cooperation Base of Biomedical Materials Technology and Application, CAS Key Laboratory of Magnetic Materials and Devices, Zhejiang Engineering Research Center for Biomedical Materials, Ningbo Institute of Materials Technology and Engineering Chinese Academy of Sciences Ningbo China; ^29^ State Key Laboratory of Molecular Vaccinology and Molecular Diagnostics & Center for Molecular Imaging and Translational Medicine, School of Public Health Xiamen University Xiamen China; ^30^ Laboratory of Chemical Biology and State Key Laboratory of Rare Earth Resource Utilization, Changchun Institute of Applied Chemistry Chinese Academy of Sciences Changchun Jilin China; ^31^ Key Laboratory of Biomass Chemical Engineering of Ministry of Education, Center for Bionanoengineering, and Department of Chemical and Biological Engineering Zhejiang University Hangzhou China; ^32^ CAS Key Laboratory of Nano‐Bio Interface, Division of Nanobiomedicine and i‐Lab, Suzhou Institute of Nano‐Tech and Nano‐Bionics Chinese Academy of Sciences Suzhou China; ^33^ Department of Civil and Environmental Engineering and Center for Environmental Implications of Nano Technology (CEINT) Carnegie Mellon University Pittsburgh Pennsylvania USA; ^34^ National Key Laboratory of Plant Molecular Genetics, Chinese Academy of Sciences Center for Excellence in Molecular Plant Sciences, Institute of Plant Physiology and Ecology, Shanghai Institutes for Biological Sciences Chinese Academy of Sciences Shanghai China; ^35^ Laboratory of Controllable Nanopharmaceuticals, Center for Excellence in Nanoscience and CAS Key Laboratory for Biomedical Effects of Nanomaterials and Nanosafety, National Center for Nanoscience and Technology Chinese Academy of Sciences Beijing China; ^36^ Department of Chemistry and Biochemistry The University of Texas at El Paso El Paso Texas USA; ^37^ Research Center for Functional Materials National Institute for Materials Science Tsukuba Ibaraki Japan; ^38^ Fachbereich Physik, CHyN University of Hamburg Hamburg Germany; ^39^ California NanoSystems Institute, Department of Chemistry and Biochemistry, Department of Bioengineering, and Department of Materials Science and Engineering University of California Los Angeles California USA; ^40^ School of Chemistry University of New South Wales Sydney New South Wales Australia; ^41^ Frontiers Science Center for Transformative Molecules, School of Chemistry and Chemical Engineering Shanghai Jiao Tong University Shanghai China; ^42^ The Florey Department of Neuroscience and Mental Health The University of Melbourne Melbourne Victoria Australia; ^43^ Key Laboratory of Animal Immunology of the Ministry of Agriculture, Henan Provincial Key Laboratory of Animal Immunology Henan Academy of Agricultural Sciences Zhengzhou China; ^44^ School of Plant Sciences and Bio5 Institute University of Arizona Tucson Arizona USA; ^45^ Shenzhen Institute of Aggregate Science and Technology, School of Science and Engineering The Chinese University of Hong Kong Shenzhen China

**Keywords:** nanocarrier, plant nano‐biotechnology

## Abstract

Biological applications of nanomaterials as delivery carriers have been embedded in traditional biomedical research for decades. Despite lagging behind, recent significant breakthroughs in the use of nanocarriers as tools for plant biotechnology have created great interest. In this Perspective, we review the outstanding recent works in nanocarrier‐mediated plant transformation and its agricultural applications. We analyze the chemical and physical properties of nanocarriers determining their uptake efficiency and transport throughout the plant body.

## Graphical abstract

This perspective reviews and explores the customized design of “smart” nanocarriers for revolutionizing plant biotechnology in transformation and in agricultural applications, and discusses the challenges and tactical approaches for adapting biomedical nanocarriers for these purposes

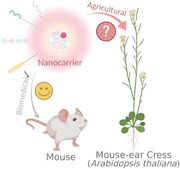

Applications of nanotechnologies have pervaded almost every aspect of scientific research. Nanodelivery vehicles have demonstrated many advantages over traditional macromolecular carriers, due to their flexible sizing, composition, physical properties, and surface chemistry. Biomedical applications of nanoparticles have been successfully translated into clinical procedures.^[^ ^1^, ^2^ ^]^ In contrast, while there are important initial studies, applications in plant biotechnology have lagged behind.^[^ ^3^ ^]^ However, not all innovations that have been used in the field of nanoparticle‐based nanomedicine have yet been applied in plant biotechnology. In this perspective, we offer a vision of what might be possible in the near future. Here, “nanocarriers” are defined as biomolecular/agrochemical delivery vehicles. We focus on current progress and gaps in developing nanoscale carrier applications for plants, consider the challenges and potential solutions, and conclude by exploring controlled cargo release for plant nanocarriers.

## 1 THE DAWN OF A REVOLUTION IN PLANT BIOTECHNOLOGY LED BY NANOCARRIERS

Successful delivery of biomolecules into plants could underpin game‐changing opportunities to revolutionize agriculture by changing and facilitating plant breeding as well as by optimizing agricultural practices.

### 1.1 Plant gene transformation in the nanotechnology era

Genetic engineering in plants has long been a challenging exercise. Current biomolecule delivery systems into the plant cells still are hampered by shortcomings and drawbacks. For instance, *Agrobacterium*‐mediated transformation imposes limits on the size of the insertable foreign gene and on the range of plant species susceptible to transformation.^[^ ^4^ ^]^ Particle bombardment overcomes the selectivity of the *Agrobacterium* species bottleneck.^[^ ^4^ ^]^ However, this technology is constrained by elevated costs and extremely low transformation efficiencies. Both systems are further limited by the subsequent challenging and laborious work of tissue culture when direct genetic modification of germline cells cannot be performed.^[^ ^4^ ^]^ Novel application of nanocarrier‐mediated transformation concepts may offer an exciting new approach to overcome these hurdles. There are tremendous opportunities and value to developing these capabilities.^[^ ^5^ ^]^

**FIGURE 1:**
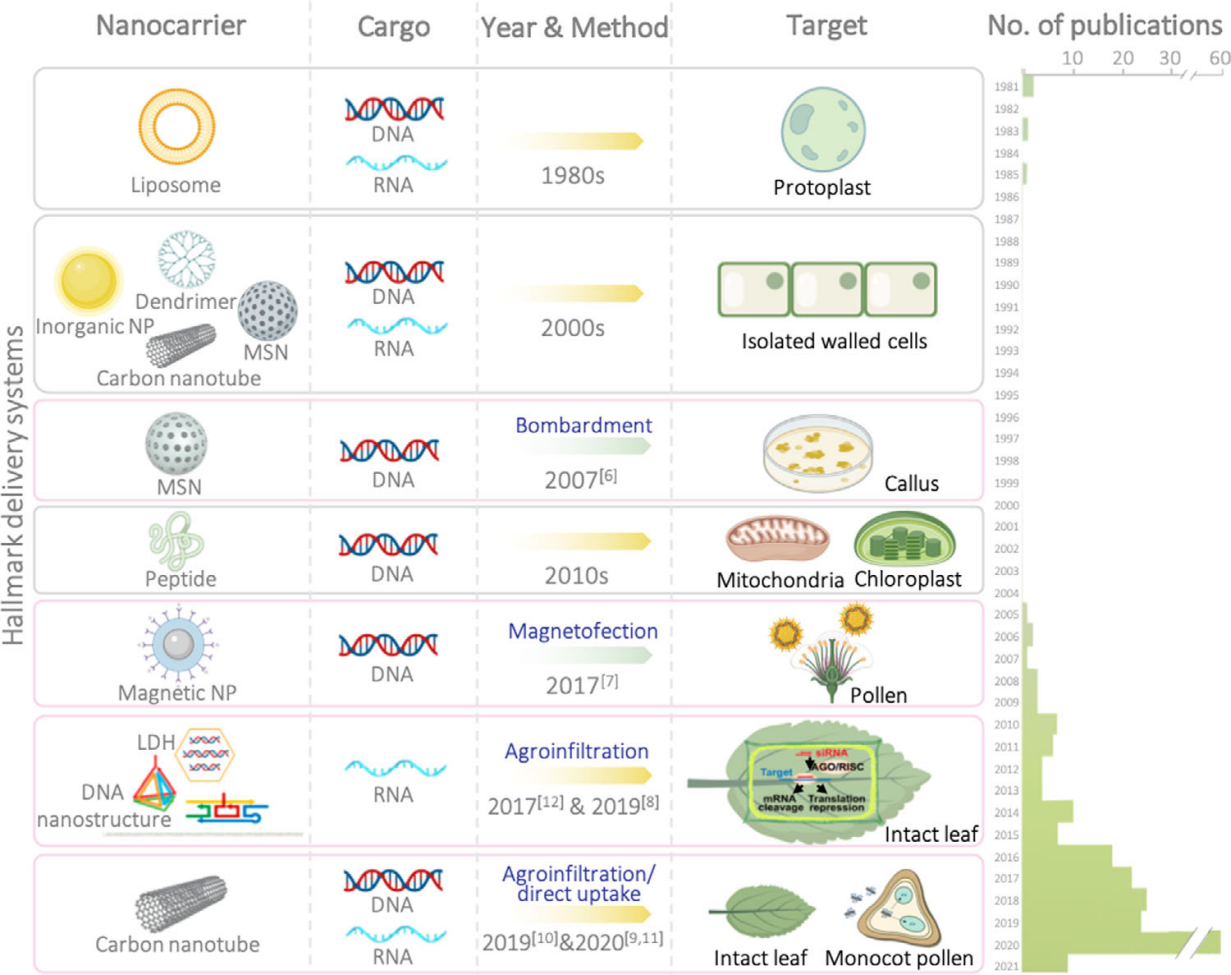
Schematic showing the number of research articles and reviews appearing in regard to nanomaterials employed for plant gene transformation, searched with the key terms “nanoparticles” AND “plant transfection” OR “plant transformation” in Google Scholar, and schematic illustrations of key milestones concerning the respective delivery systems (pink boxes). Transformation events (arrows) are color‐coded (i.e., yellow: transient transformation; moss green: stable transformation). Reference citations are provided for each milestone study. Abbreviations: layered double hydroxide clay nanosheet (LDH); mesoporous silicon nanoparticle (MSN); nanoparticle (NP).^[^ ^6^, ^7^, ^8^, ^9^, ^10^, ^11^, ^12^ ^]^ Figure created with BioRender.com

Nanocarrier‐mediated gene modification in plants was first introduced in the early 1980s using protoplasts in which cell walls are absent,^[^ ^13^ ^]^ for which liposomes were one of the first attempted and have been amongst the most investigated nanocarriers (Figure 1). Successful transformation was reported for tobacco,^[^ ^13^, ^14^ ^]^ carrot,^[^ ^15^ ^]^ and maize.^[^ ^16^ ^]^ Later, a number of studies exploited isolated turfgrass cells and tobacco BY‐2 cells, where the cell wall remains present, realizing transient gene transformation mediated by polymeric dendrimers, carbon nanotubes, or ultrasonic‐aided ZnS nanoparticles (Figure 1).^[^ ^17^ ^]^ In these cases, successful penetration across the cell wall was achieved by several types of nanocarriers with a range of different sizes and charges, yet no underlying mechanism has been reported.

Only very recently has direct genome transformation of intact plant tissue has been achieved. Here, transient transformation has been more readily accomplished compared to stable transformation (Table 1). Successful transient engineering of model plants, including Arabidopsis and tobacco, was mediated by direct internalization of nanocarriers such as carbon nanotubes,^[^ ^10^, ^11^ ^]^ cationic polymeric nanoparticles,^[^ ^18^ ^]^ and layered double hydroxide clay nanosheets (Figure 1).^[^ ^8^ ^]^ Species limitations were also overcome through the use of carbon nanotubes as well as DNA nanostructures, extending applications of nanocarriers to economic crops such as cotton and wheat.^[^ ^9^, ^11^ ^]^

**TABLE 1:** Summary of nanocarrier‐mediated plant gene transformation vehicles

Nanocarrier	Transformation	Cargo	Nanocarrier size (nm)	Plant species	Plant tissue	Cell wall	External aid
MAL‐PEG‐PLL^[^ ^19^ ^]^	T	pDNA	90 ± 3	*Nicotiana benthamiana*	Leaf	Y	N
DNA nanostructure^[^ ^9^ ^]^	T	siRNA	At least one dimension ≦ 10	*Nicotiana benthamiana*	Leaf	Y	N
Layered double hydroxide clay nanosheets^[^ ^8^ ^]^	T	dsRNA	80–300	*Arabidopsis thaliana* *Nicotiana tabacum*	Leaf	Y	N
Chitosan NPs^[^ ^20^ ^]^	T	pDNA	86.8 ± 2.6	*Lycopersicon esculentum*	Leaf	Y	N
Single/multi‐walled carbon nanotube (SWNT/MWNT)^[^ ^10^, ^11^ ^]^	T	dsDNA siRNA	At least one dimension ≦ 20	*Nicotiana benthamiana* *Eruca sativa* *Triticum aestivum* *Gossypium hirsutum*	Leaf protoplast	Y&N	N
Cationic dendrimer^[^ ^18^ ^]^	S	DNA dsRNA	52.33 ± 5.04	*Arabidopsis thaliana*	Root cell	Y	N
Arg‐SWNT^[^ ^21^ ^]^	T	GFP‐plasmid DNA	≤320	*Nicotiana tabacum*	Root	Y	Enzymatic cell wall loosening and ligand
CaP^[^ ^22^ ^]^	S (TC required)	pDNA	20–50	*Brassica juncea*	Hypocotyl explant	N	Ultrasound
Starch NP^[^ ^23^ ^]^	T	pDNA	50–100	*Dioscrea zigiberensis*	Callus	Y	Ultrasound
Magnetic NPs^[^ ^7^ ^]^	S	pDNA	200	*Gossypium hirsutum* *Capsicum annuum* *Cucurbita moschata* *Cucurbita pepo* *Lilium brownii*	Pollen	Y	Magnetic field
Poly(phenylene ethynylene)^[^ ^24^ ^]^	S (TC required)	siRNA	60–80	*Nicotiana tabacum*	Protoplast	N	N

Abbreviations: N: no; NP: nanoparticle; TC: tissue culture; T: transient; S: stable; Y: yes.

Nevertheless, the majority of stable transformation studies employing nanocarriers require subsequent explant regeneration (Table 1). One important stable transformation study employed bombardment of tobacco callus with mesoporous silica nanoparticles.^[^ ^6^, ^25^ ^]^ Other mechanically aided applications included vortexing/oscillation‐assisted silicon carbide whiskers that expanded the application to monocot species.^[^ ^26^ ^]^ Direct uptake of foreign biomolecules was only reported for relatively small nanocarriers (20–50 nm), including plasmid DNA loaded by calcium phosphate (CaP) into *Brassica juncea* L. hypocotyls (Table 1).^[^ ^22^ ^]^

Ready production of transgenic plants bypassing tissue culture has involved direct gene manipulation of germline cells. The pioneering study by Zhao et al. reported use of pollen magnetofection^[^ ^7^ ^]^ for eudicot species, including cotton (*Gossypium hirsutum* Linn.), pepper (*Capsicum annuum* L.), pumpkin (*Cucurbita moschata*), cocozelle (*Cucurbita pepo* L.), and the monocot species, lily (*Lilium brownii*). However, reproducible results remain elusive, at least in monocot plants, with this technique.^[^ ^27^ ^]^ More recently, direct penetration through pollen exines has been reported for carbon nanotubes (Figure 1). However, subsequent stable transformation was not reported, suggesting that the foreign green fluorescence protein gene was successfully introduced into the cytoplasm, but without achieving integration into the genome.^[^ ^12^ ^]^

During the delivery process, nanocarriers not only benefit cellular penetration, but also can extend biomolecule longevity (e.g., up to 30 days for effective siRNA release^[^ ^8^ ^]^) by providing protection from nucleases.^[^ ^10^ ^]^ As a result, substantially increased transformation efficiencies are consistently reported for nanocarrier‐mediated approaches. Thus, Naqvi et al. found ∼80% transformation efficiency using plasmid DNA loaded by CaP nanoparticles compared favorably to ∼55% using *Agrobacterium tumefaciens* and to 8% using naked plasmid DNA.^[^ ^22^ ^]^ In another study, the stable pollen magnetofection technique recorded a success rate of 2–12% in obtaining transgenic seeds.^[^ ^7^ ^]^ Again, a 95% gene silencing efficiency was reported at the mRNA level by carbon nanotube delivered siRNAs.^[^ ^10^ ^]^ In addition, the precision and efficiency of nanocarrier‐delivered biomolecules can reduce the minimal effective amounts of loaded biomolecules by factors of up to 1000‐fold.^[^ ^6^ ^]^ Therefore, the highly efficient transformation enabled by nanocarriers should accelerate progress in plant biotechnology.

### 1.2 Advantages of nanocarriers over traditional agrochemical application

Another application for nanocarriers is in the delivery of agrochemicals and fertilizers.^[^ ^28^, ^29^ ^]^ Traditional methods of application of agrochemicals and fertilizers have plagued the agricultural industry with significant disadvantages, including overdosing, bioaccumulation, growing resistance, air/water pollution, non‐specific targeting, and disruption of biosphere microorganism communities. Use of nanomaterials as carriers offers new possibilities for efficient and targeted delivery of a range of agrochemicals including fertilizers and pesticides,^[^ ^30^ ^]^ and agents to manage abiotic stress from climate change.^[^ ^31^ ^]^ For instance, the efficacy of targeting nematodes has been enhanced by superior terrestrial diffusion and tissue penetration capacities of tobacco virus‐based nanoparticles.^[^ ^32^ ^]^ In addition, nanocarrier‐applied fertilizer has significantly enhanced crop productivity compared to traditional approaches.^[^ ^33^, ^34^, ^35^ ^]^ A recent outstanding review by Kumar et al. provides valuable insights into nanocarrier‐mediated plant pest and disease resistance.^[^ ^36^ ^]^

## 2 PATHWAYS FOR NANOPARTICLE DELIVERY IN PLANTS AND THE CHALLENGES THEY POSE

Beyond applications, the *in planta* uptake and transport behavior of nanocarriers are poorly understood. Here, we explore the relationships between the efficacy of nanoparticle uptake and transport with their chemical and physical properties. Much of our knowledge derives from studies in environmental sciences relating to the uptake and translocation tracing of solid nanoparticles,^[^ ^37^, ^38^ ^]^ but some studies have used polymeric carriers similar to those used in biomedicine^[^ ^31^ ^]^ and peptide‐based nanocarriers.^[^ ^39^ ^]^

Delivering nanomaterials into plant cells and targeting them to specific organelles such as chloroplasts is challenging. Routes of entry are generally limited to either root uptake from soil or through either cuticular or stomatal uptake pathways after foliar application. Biological barriers to uptake such as plant cuticle, epidermis, and cell walls all present barriers to uptake. Once a nanoparticle has passed these barriers, movement through mesophyll or plant vasculature presents additional barriers to transport, for example, sieve plates in phloem. These barriers to transport, and the impact of the nanocarrier properties on their ability to cross barriers and target specific locations in plants are beginning to be explored, but this remains a research area that can leverage advancements made in biomedicine after addressing some of the key challenges outlines below.^[^ ^37^, ^40^, ^41^ ^]^

### 2.1 Cellular level uptake of nanoparticles

#### 2.1.1 Extracellular compartment entry

**FIGURE 2:**
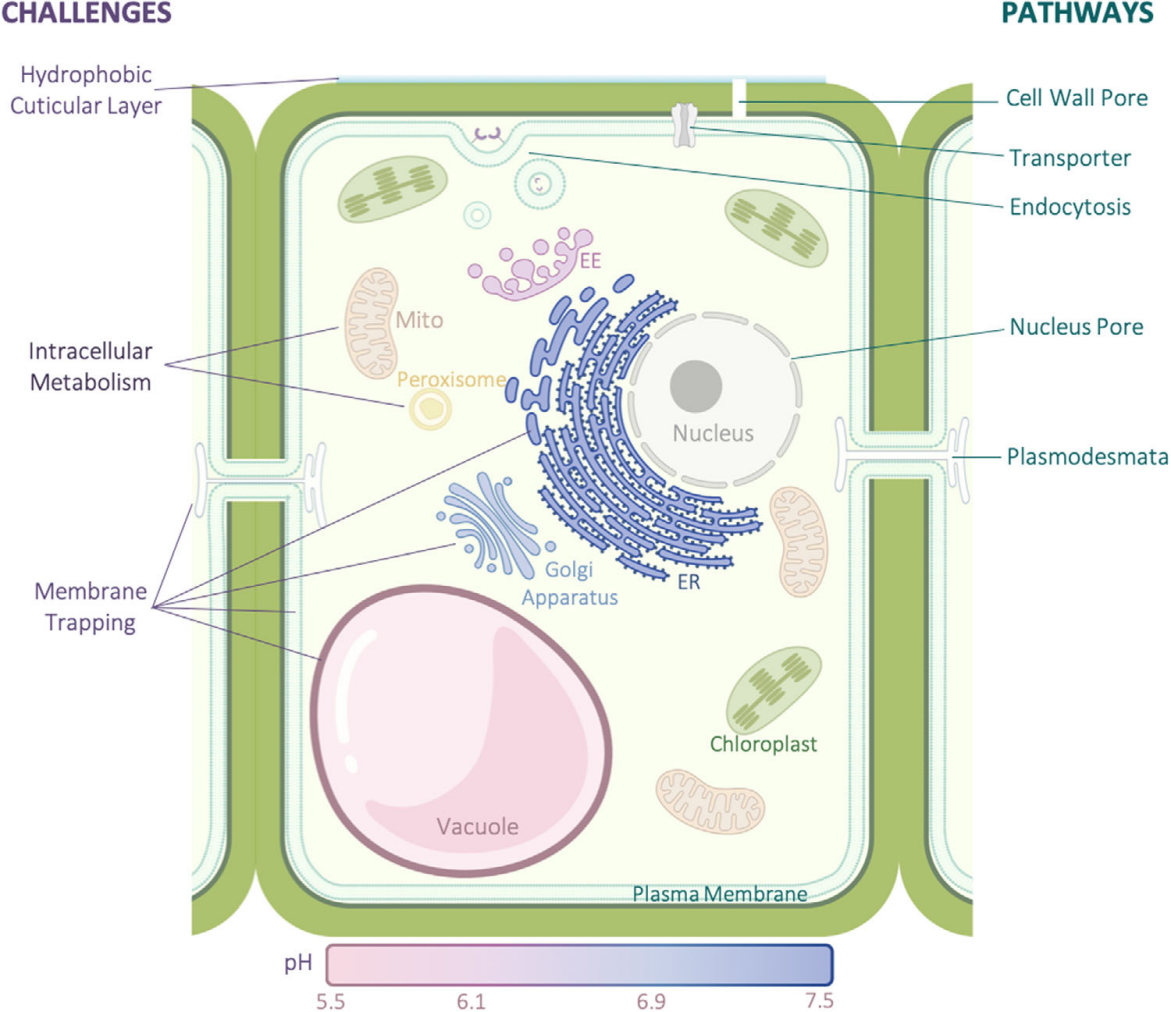
Challenges (dark purple font) for nanocarrier uptake into plant cells and feasible pathways (dark green font) facilitating epidermal and cellular transport. The colored bar corresponds to the known pH in endocytotic organelles. Abbreviations: early endosome (EE); endoplasmic reticulum (ER); mitochondria (Mito). Figure created with BioRender.com

The most obvious difference between animal and plant cells is the presence of the plant cell wall (Figure 2) that creates the most conspicuous challenge to nanocarrier delivery into plant cells. Despite the characterization of cell wall pores ranging in size from 3 to 15 nm,^[^ ^42^ ^]^ no size limitation has been confirmed for nanoparticle transport across the cell wall. Other than a few cases of size‐selection for gold nanoparticles (AuNPs),^[^ ^43^ ^]^ nanocarriers up to 100 nm in diameter were reported as being transported through cell walls (Table 2). In addition, the various shapes of nanoparticles seem to have little effect on their transport efficiencies across cell walls, and we can find no published studies that have demonstrated uptake mechanisms beyond direct penetration by DNA nanostructures and carbon nanotubes.^[^ ^9^, ^11^ ^]^

**TABLE 2:** Summary of nanoparticles translocation through plant vascular systems in relation to their size and surface properties

Nanoparticles	Size (nm)	Surface charge	Plant species	Mono (M) or eudicot (E)	Vascular translocation route	NPs application site
Liposome^[^ ^35^ ^]^	100	Not indicated	*Solanum lycopersicum*	E	Phloem	Source leaf to sink root
PAA‐*b*‐PNIPAm^[^ ^31^ ^]^	13, 17, 29, 32	−ve	*Solanum lycopersicum*	E	Phloem	Leaf
TiO_2_^[^ ^44^ ^]^	35	Not indicated	*Aristolochia debilis*	E	Xylem	Root
AgNPs^[^ ^45^ ^]^	20/40/80	+ve	*Arabidopsis thaliana*	E	Xylem	Root
AgNO_3_^[^ ^46^ ^]^	6.3–7.8	−ve	*Landoltia punctata*	M	Xylem–Phloem	Root
Carbon coated iron NPs^[^ ^47^ ^]^	∼50	+ve/neutral	*Cucurbita pepo*	E	Xylem	Injection into leaf petiole pith cavity, magnetic guide; or foliar spray
Cr_2_O_3_^[^ ^48^ ^]^	50	−ve	*Glycine max*	E	Xylem–Phloem–Phloem	Root
(1) ZnO (2) CuO (3) CeO_2_^[^ ^49^ ^]^	1. 30–40 2. 25–55 3. 30–50	Not indicated	*Daucus carota*	E	Xylem–Phloem–Phloem	Root
TiO_2_^[^ ^50^ ^]^	30	Not indicated	*Zea mays*	M	Xylem–Phloem–Phloem	Root
Carbon nanotube^[^ ^51^ ^]^	35	Not indicated	*Brassica juncea*	E	Xylem	Root
Silica NPs^[^ ^52^ ^]^	20	−ve	(1) *Arabidopsis thaliana* (2) *Triticum aestivum* (3) *Lupinus angustifolious*	(1) E (2) M (3) E	Xylem	(1) Whole seedling (2) Root (3) Root
C_70_^[^ ^53^ ^]^	239.7	Not indicated	*Oryza sativa*	M	Xylem (Phloem participation required as since germination all organs are essentially sink tissue)	Seed germination
CeO_2_^[^ ^54^ ^]^	(1) 12.0 ± 3.4 (2) 19.4 ± 5.7 (3) 14.5 ± 3.3	(1) +ve (2) Neutral (3) −ve	*Triticum aestivum*	M	Xylem–Phloem–Phloem	Roots
AuNPs^[^ ^55^ ^]^	6–10	+ve and −ve	(1) *Oryza sativa* (2) *Lolium perenne* (3) *Raphanus sativus* (4) *Cucurbita mixta*	(1) M (2) M (3) E (4) M	Xylem	Root
Silica NPs^[^ ^56^ ^]^	20	−ve	(1) *Arabidopsis thaliana* (2) *Triticum aestivum* (3) *Lupinus angustifolious* (4) *Zea mays*	(1) E (2) M (3) E (4) M	(1) Not indicated (2) Xylem (3) Xylem (4) Xylem	Root
CeO_2_^[^ ^57^ ^]^	22.6 ± 20.9	+ve	*Cucumis sativus*	E	Xylem and Phloem	Root
CuO^[^ ^58^ ^]^	20–40	−ve	*Zea mays*	M	Xylem and Phloem	Root
AuNPs^[^ ^59^ ^]^	15, 25, and 50	Not indicated	Hybrid poplar plants	E	Xylem and Phloem	Root and leaf
Nd_2_O_3_^[^ ^60^ ^]^	30–45, agglomerated 448.3 ± 1.0	+ve then to −ve	*Cucurbita maxima*	E	Xylem and Phloem	Root
AuNPs^[^ ^61^ ^]^	(1) 30–90; (2) 35	(1) +ve (2) −ve	*Citrullus lanatus*	E	Phloem	Leaf
AuNPs^[^ ^62^ ^]^	3.5, 12, and 50	−ve	*Triticum aestivum*	M	Phloem	Leaf

Abbreviations: E: eudicotyledon; M: monocotyledon; NP: nanoparticle.

It seems that limitations for cell wall transport more likely arise from general physicochemical characteristics of the nanoparticles. Notably, alterations to their surface coatings have been shown to modulate ease of entry and/or translocation. Examples include cell wall penetration by 50 nm AuNPs, coated with citrate or polyvinylpyrrolidone^[^ ^62^ ^]^ and by 46 nm carbon‐coated iron nanoparticles.^[^ ^47^ ^]^ Moreover, some nanoparticles are reportedly capable of inducing the formation of larger pores in cell walls.^[^ ^63^ ^]^ Such enlargement has been suggested to result from as yet unknown interactions between the surface coatings of nanoparticles and cross‐linking pectins controlling pore sizes in cell walls.^[^ ^63^, ^64^, ^65^ ^]^ In addition, stiffness and compactness of DNA nanostructure nanocarriers have been suggested to contribute to their internalization.^[^ ^9^ ^]^ Whether these factors are associated only with direct penetration or with undiscovered uptake mechanisms requires further exploration.

In the case of plant aerial epidermal cells, the presence of a dense, hydrophobic extracellular cuticular layer introduces an additional trapping challenge for nanocarrier uptake.^[^ ^66^ ^]^ Potential entry routes of nanocarriers into leaves via both cuticular pathways and stomatal pathways have recently been reviewed.^[^ ^37^ ^]^ There is ample evidence for stomatal uptake of nanoparticles, for example, hydrophilic chitosan nanocarriers (86.8 nm in size) entered through stomata in the plant leaves.^[^ ^20^ ^]^ However, there is also evidence that Au nanoparticles with amphiphilic coatings can also be taken up directly through the cuticle.^[^ ^62^ ^]^ Indeed, other nanocarriers, both hydrophilic and hydrophobic, also gain entry into plant leaves (Tables 1 and 2). While conventional thinking suggests stomata (from 19.1 to 71.5 µm in diameter^[^ ^67^ ^]^) should represent the most significant pathway into the leaves, other less explored entry routes are reported in the literature including cuticle, trichomes, hydathodes, necrotic spots.^[^ ^62^ ^]^ The size exclusion limits for these different routes of entry, and the properties of the nanocarriers affecting uptake still need to be determined. For example, the limits of stomatal passage have only been reported for a 43 nm carboxylate‐modified surface nanoparticle and not for its 1.1 µm analogue.^[^ ^68^ ^]^ These results suggest the existence of selectivity in the stomatal pathway.

Direct penetration of the plasma membrane has been reported for both single‐walled carbon nanotubes (SWNTs)^[^ ^11^ ^]^ and multi‐walled carbon nanotubes (MWNTs),^[^ ^69^ ^]^ and is followed by plasma membrane repair.^[^ ^69^ ^]^ Other likely pathways for nanomaterial transport across the plasma membrane include endocytosis. Unlike the endosomal escape challenge proposed during endocytotic uptake of nanocarriers into animal cells,^[^ ^70^, ^71^ ^]^ plant cells retain their acidic microenvironment in the trans‐Golgi network (pH 6.1), followed by alkaline pre‐vacuolar compartments and finally an acidic vacuole (pH 5.5–6).^[^ ^72^ ^]^ Thus, variations of pH‐responsive cargo release used in nanomedicine will require testing in plant cells in terms of their *in vivo* fate.

#### 2.1.2 Intracellular microenvironment

Once within the cytoplasm, nanocarriers face further challenges. These can be primarily categorized as being a consequence of membrane trapping by organelles, and of interactions with the intracellular microenvironment.

Trapping of nanoparticles in organelle membranes could cause cytotoxicity or limit nanocarrier bioavailability (Figure 2). SWNTs were reported to be irreversibly trapped in chloroplast membranes^[^ ^41^, ^73^ ^]^ and MWNTs, with lengths less than 100 nm, were trapped by membranes of vacuoles, plastids, and nuclei.^[^ ^69^ ^]^ The mechanism(s) contributing to membrane trapping remains to be elucidated; one plausible mechanism proposed involves ionic binding to negatively charged membrane surfaces. In this context, effective membrane permeability was achieved using a range of positively charged nanoparticles.^[^ ^18^, ^21^ ^]^ An insightful model by Kwak et al. predicted that passive transport of nanoparticles through negatively charged membranes is largely regulated by their size and surface charge.^[^ ^74^ ^]^ However, this model is based on only a few types of nanocarriers and coatings. Recent work has shown that biorecognition molecules decorated onto the nanocarrier can help to guide them to specific organelle membranes, for example, chloroplasts.^[^ ^40^ ^]^

The complexity of the intracellular microenvironment presents another challenge. Nanocarriers are foreign objects that can trigger an innate immune response in plants (Figure 2).^[^ ^75^ ^]^ For instance, exposure to Cu(OH)_2_ nanoparticles caused elevated polyamine levels.^[^ ^76^ ^]^ Overproduction of reactive oxygen species (ROS) levels, coupled with defense‐related antioxidant activities, were detected in Arabidopsis upon exposure to CeO_2_ and In_2_O_3_ nanoparticles,^[^ ^57^, ^77^ ^]^ in Nd_2_O_3_‐treated pumpkin (*Cucurbita maxima*),^[^ ^60^ ^]^ and in CuO‐treated wheat.^[^ ^78^ ^]^ Thus, fabrication of an ideal nanocarrier for intracellular delivery should include materials that minimize triggering defense responses.

Encouragingly, aside from acting as a delivery agent, nanocarriers can also protect their cargos from intracellular nucleases by reducing exposure of the free ends of the DNA/RNA cargo when conjugating biomolecules onto nanocarriers.^[^ ^10^, ^11^ ^]^ Studies *in vitro* have shown that the binding of siRNAs to carbon nanotubes reduced their degradation by RNases from 98% to 16%.^[^ ^10^ ^]^

#### 2.1.3 Intracellular organelles

Surface modification of nanocarriers with specific ligands offers exciting opportunities for their entry into intracellular compartments (Figure 2), as they are able to carry cargos of molecular weight several times their own. Some ligands carrying nucleic acid cargoes have been designed for selective targeting into nuclei (virE2),^[^ ^79^ ^]^ mitochondria, or chloroplasts.^[^ ^80^ ^]^ A library of cell‐penetrating peptides (CPPs) was recently screened for incorporation into BY‐2 cells and leaves of Arabidopsis, tobacco, tomato, poplar, and rice.^[^ ^81^ ^]^ The transport efficiency is independent of energy, temperature, or receptor,^[^ ^79^ ^]^ but rather is determined by the amount of CPPs located at the surface of the complex.^[^ ^82^ ^]^ Interestingly, the efficiency of CPP‐mediated transport differs between monocots and eudicots (Table 1),^[^ ^81^ ^]^ while the underlying mechanisms remain unknown.

### 2.2 *In planta* transport of nanocarriers

The scale of industrial agriculture requires applications of nanomaterial‐mediated drug/fertilizer at to the crop canopy or to soil/root sites.^[^ ^83^ ^]^ Previous sections reviewed nanocarrier uptake into cells. However, agricultural application depends heavily on nanoparticles spreading from their initial uptake sites over short distances between cells and over longer distances within the plant vascular system. The factors influencing these transport processes, both due to plant physiological activity or the engineered nanocarrier properties are poorly understood.

#### 2.2.1 Short distance transport

**FIGURE 3:**
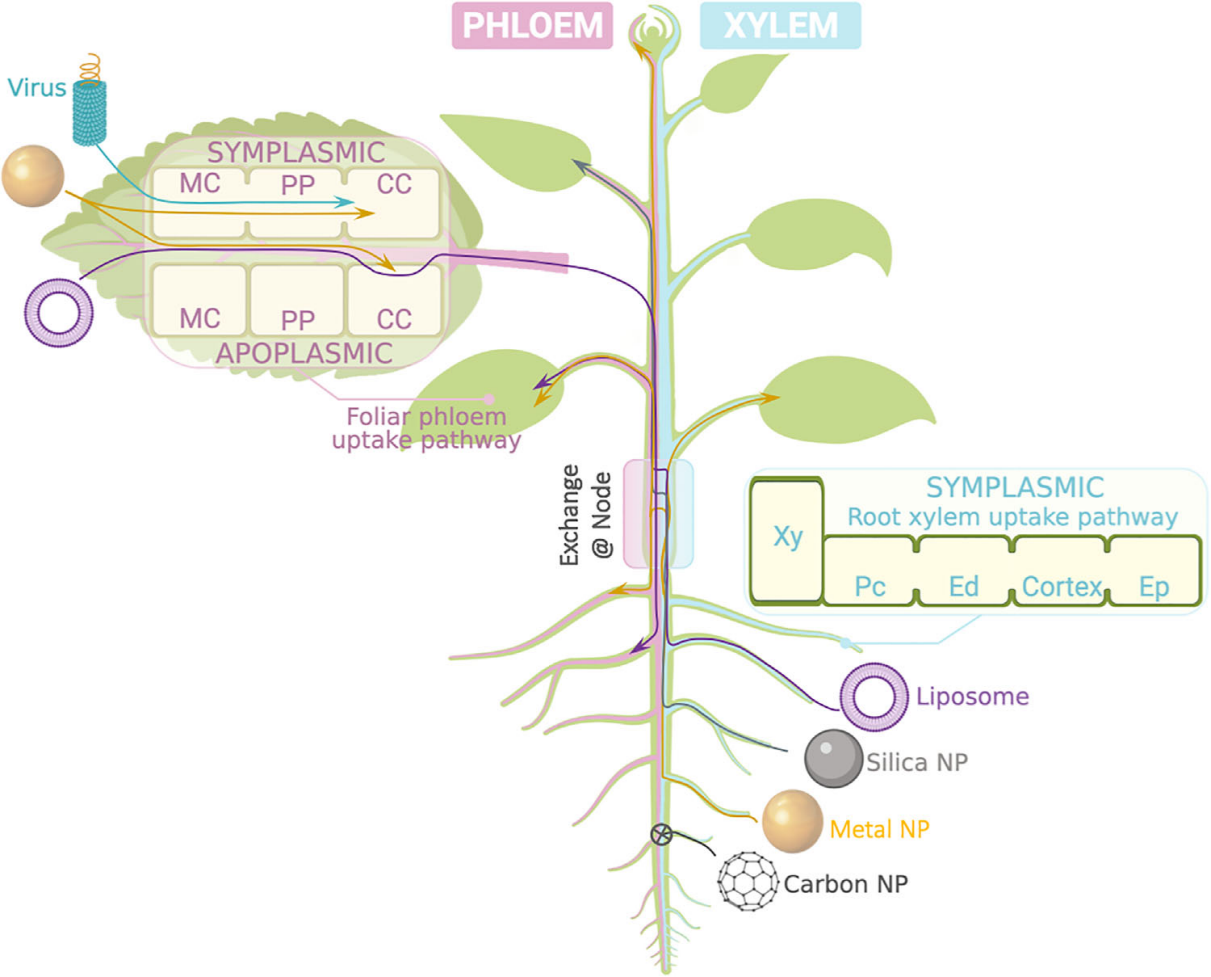
*In planta* uptake and vascular transport of nanoparticles. Abbreviations: Companion cell (CC); endodermis (Ed); epidermis (Ep); mesophyll cell (MC); nanoparticle (NP); pericycle (Pc); phloem parenchyma cell (PP); xylem (Xy). Figure created with BioRender.com

Short distance transport between cells occurs via symplasmic (cytoplasm to cytoplasm through interconnecting plasmodesmata) or apoplasmic (cell wall/extracellular space) pathways (Figure 3).^[^ ^84^ ^]^ Nanoparticle trapping can occur around plasmodesmal collars independent of their size (AuNPs;^[^ ^59^ ^]^ silver nanoparticles (AgNPs)^[^ ^45^ ^]^). This trapping may be caused by a combination of plasmodesmal selective transport and passive transfer of nanoparticles through plasmodesmata being limited to small molecular exclusion limits (∼3 nm).^[^ ^85^ ^]^ Ameliorating this bottleneck could be achieved by exploiting plant viruses that encode specialized movement proteins that dilate plasmodesmata.^[^ ^85^, ^86^ ^]^ For example, movement protein TGBp1, from potato virus X, increased the size exclusion limit of plasmodesmata to 41 nm.^[^ ^87^ ^]^ Thus, these successes in engineered nanoparticles promise opportunities to enhance their transport through symplasmic pathways (Figure 3).

#### 2.2.2 Vascular transport

For foliar/soil‐applied nanoparticles designed to deliver nutrients or pest protection agents to the entire plant, their translocation via a vascular transport route is key to realizing their agricultural potential. However, no patterns can be discerned from the results of the limited current studies available. The translocation routes followed by nanoparticles are likely to be determined by their surface charge and chemical properties.

Xylem transport of mesoporous silica,^[^ ^56^ ^]^ or metal‐based (e.g., Au;^[^ ^59^ ^]^ cerium;^[^ ^58^ ^]^ TiO_2_^[^ ^63^ ^]^) nanoparticles (Figure 3 and Table 2) was facilitated by being bound to chelators such as ethylenediaminetetraacetic acid.^[^ ^55^ ^]^ Surface charge of metal‐based nanoparticles also affects their transport efficacy. For instance, negatively charged nanoparticles were efficiently transported from roots to shoots in the xylem.^[^ ^54^, ^55^ ^]^

Fewer details have been reported regarding phloem translocation of nanoparticles compared to xylem transport (Table 2 and Figure 3). However, evidence indicates that phloem loading after foliar application is possible for both metal NPs and polymeric carriers, with up to 30% of foliar‐applied Au NPs moving via phloem from leaf to roots^[^ ^62^ ^]^ and up to 50% of applied PAA‐*b*‐PNiPAm starry polymers applied to leaves translocating to non‐exposed plant tissues.^[^ ^31^ ^]^ Phloem loading of labeled glycine methyl ester‐conjugated polysuccinimide nanoparticles was observed in banana plants.^[^ ^39^ ^]^ In the case of liposomes, bidirectional phloem transport was reported for both leaves and roots.^[^ ^35^ ^]^ One of the few papers addressing the anatomy of phloem transport suggested that, following foliar application, AuNPs moved symplasmically from mesophyll cells to the phloem (Figure 3).^[^ ^61^ ^]^ Phloem mobility of potato virus X‐based nanoparticles was affected by the presence of tryptophan in, and the isoelectric point of, the surface peptide.^[^ ^87^ ^]^

Translocation via both vascular pathways has also been reported (Table 2). For instance, metal‐based nanoparticles taken up via the root xylem have been frequently observed in shoot apices (Table 2 and Figure 3). This uptake must involve xylem/phloem exchange events in nodes.^[^ ^88^ ^]^ Such events would require nanoparticles to penetrate multiple layers of cell walls and to cross several plasma membranes; both processes are dependent on nanoparticle size and surface properties, as discussed previously.

In addition to the nanocarrier properties, plant physiology (e.g., transpiration rate) will likely affect uptake and translocation. There is some evidence for different transport characteristics for identical nanoparticles in monocots and eudicots (Table 2). For example, dicots in general took up and translocated more CeO_2_ nanoparticles from roots to shoots than monocots, regardless of charge.^[^ ^89^ ^]^ Using radiolabeled MWNTs, Zhao et al. traced their movement in *A. thaliana*, soybean (*Glycine max*), maize, and rice (*Oryza sativa* L.).^[^ ^90^ ^]^ MWNT transport in the eudicots (*A. thaliana* and soybean) was nearly 1.5‐ to 3‐fold greater than in the monocots (rice and maize), although the underlying cause remains unknown. Moreover, translocation rates of nanocarriers applied to roots are often low in monocots (usually < 1%), suggesting that xylem transport is less efficient for nanocarriers for this plant physiology than phloem transport.

An unexplored area that may be affecting the transport of nanocarriers in planta is the formation of a protein corona on the carriers. Similar to the protein corona formed on nanomaterials in animals, nanocarriers in plant cytosol should form a protein corona. Cytosol contains an abundance of proteins (e.g., glutamate) that will likely interact with the nanocarrier and affect its physicochemical properties. A protein corona developed on CuO nanoparticles exposed to pumpkin xylem fluid.^[^ ^91^ ^]^ The types of proteins that form on the nanocarriers, and the influence of this corona on transport in plants and physiological response to nanocarriers needs to be better understood to promote rational design of effective carriers.

## 3 EXPLORATION OF TARGETED DELIVERY AND CARGO RELEASE FROM NANOCARRIERS IN PLANTS

In addition to uptake and transport, successful release of cargos into designated compartments is of great importance for “smart” nanocarriers. Just like the special microenvironment of cancer‐tissue‐inspired designs of tumor‐specific release stimuli, all current approaches will require careful scrutiny when adapting nanocarriers for use in plants. Some approaches that have been considered to date include those based on pH gradients in cells,^[^ ^39^ ^]^ light,^[^ ^92^ ^]^ ROS,^[^ ^93^ ^]^ and temperature^[^ ^31^ ^]^ as triggering mechanisms.

The glutathione (GSH)‐triggered method of target release in cancer cell/tissue models^[^ ^94^ ^]^ appears an unlikely option for plants, as no significant concentration differences in GSH levels have been detected between plant tissues. One study employed GSH‐triggered release of nanocarrier and showed neither specific tissue nor cell targeting.^[^ ^95^ ^]^ These results are consistent with plants employing complex redox regulatory processes to maintain redox homeostasis, especially when under stress.^[^ ^96^ ^]^ On the other hand, pH‐responsiveness might be a more potent option. For instance, based on the relatively higher pH of phloem sap,^[^ ^97^ ^]^ alkaline‐triggered smart nanoparticles with minimum cytotoxicity have been designed.^[^ ^98^ ^]^ This strategy allows nanoparticle delivery of nutrients to sink tissue and pesticides to target phloem‐limited diseases, such as, citrus huanglongbing.^[^ ^99^ ^]^

External stimuli for triggered release of nanoparticle cargoes include magnetic field, ultrasound, light, and temperature.^[^ ^94^ ^]^ Light is particularly attractive for cancer therapy due to its easily regulated temporal and spatial control. There are significant challenges to identifying stimuli‐responsive materials that can be low cost, sufficiently scalable, and biocompatible enough for use in industrial agriculture.

There remains a need to develop additional innovative ways to adapt nanocarriers designed for biomedical use to agricultural applications, and this effort will require particularly thorough investigations to overcome the real differences between these systems. For instance, rather than seeking a nanocarrier that is capable of plant cell‐/tissue‐targeted release, specific promoters could be employed for the targeted introduction of foreign genes by conventional nanocarriers. Alternatively, in the case of cereal crops, their growth cycle could be exploited whereby nano‐delivery could be selectively targeted to grain filling phase alone during which vegetative growth has ceased.

## 4 CONCLUDING REMARKS AND FUTURE PROSPECTS

Opportunities for the application of nanomaterials in plants have generated great interest among plant scientists. Their flexibility, coupled to customization‐as‐per‐requirement, promises to overcome important barriers in plant biotechnology.

However, due to the fundamental structural (e.g., cell wall, plasmodesmata) and intracellular (e.g., redox homeostasis, photosynthesis) differences between plant and animal cells, laboratory and industrial application of nanoscale carriers’ entry and transport in plants requires careful testing and monitoring. This need applies especially to whether *in vivo* site‐specific targeting, delivery, and/or release would function as intended in real crop plants under environmental conditions, and, based on the current survey (Table 2), different vascular uptake patterns are likely to occur between monocot and eudicot plants. Impacts of nutrient availability, environmental conditions, growth stage, and protein corona formation *in planta* are all likely to affect the efficacy of a particular nanocarrier.

General acceptance of nanotechnology applications in agriculture will also require meticulous survey and investigation. A significant number of proposed nanocarriers require internal metabolism, especially for inorganic materials, which tend to be retained in plant cells for extended periods of time. The movement of these materials into edible parts of the plants, and their fate in crop residues and impacts on soil need to be determined. This investigation is also built upon the uncertainty among general public toward genetically modified crops and pesticide residues. Possible approaches of tracing *in vivo* nanocarriers fate include probe‐detection of nanoparticle uptake, translocation and disassociation, monitoring the levels of stress‐related genes, and testing for metabolites.

## CONFLICT OF INTEREST

We declare there are no conflict of interest.
